# A First Glimpse of Wild Lupin Karyotype Variation As Revealed by Comparative Cytogenetic Mapping

**DOI:** 10.3389/fpls.2016.01152

**Published:** 2016-07-28

**Authors:** Karolina Susek, Wojciech K. Bielski, Robert Hasterok, Barbara Naganowska, Bogdan Wolko

**Affiliations:** ^1^Department of Genomics, Institute of Plant Genetics, Polish Academy of SciencesPoznan, Poland; ^2^Department of Plant Anatomy and Cytology, University of Silesia in KatowiceKatowice, Poland

**Keywords:** polyploidization, evolution, BAC-FISH, chromosome rearrangement, lupins

## Abstract

Insight into plant genomes at the cytomolecular level provides useful information about their karyotype structure, enabling inferences about taxonomic relationships and evolutionary origins. The Old World lupins (OWL) demonstrate a high level of genomic diversification involving variation in chromosome numbers (2*n* = 32–52), basic chromosome numbers (*x* = 5–7, 9, 13) and in nuclear genome size (2C DNA = 0.97–2.68 pg). Lupins comprise both crop and wild species and provide an intriguing system to study karyotype evolution. In order to investigate lupin chromosome structure, heterologous FISH was used. Sixteen BACs that had been generated as chromosome markers for the reference species, *Lupinus angustifolius*, were used to identify chromosomes in the wild species and explore karyotype variation. While all “single-locus” in *L. angustifolius*, in the wild lupins these clones proved to be “single-locus,” “single-locus” with additional signals, “repetitive” or had no detectable BAC-FISH signal. The diverse distribution of the clones in the targeted genomes suggests a complex evolution history, which possibly involved multiple chromosomal changes such as fusions/fissions and repetitive sequence amplification. Twelve BACs were sequenced and we found numerous transposable elements including DNA transposons as well as LTR and non-LTR retrotransposons with varying quantity and composition among the different lupin species. However, at this preliminary stage, no correlation was observed between the pattern of BAC-FISH signals and the repeat content in particular BACs. Here, we describe the first BAC-based chromosome-specific markers for the wild species: *L. cosentinii, L. cryptanthus, L. pilosus, L. micranthus* and one New World lupin, *L. multiflorus*. These BACs could constitute the basis for an assignment of the chromosomal and genetic maps of other lupins, e.g., *L. albus* and *L. luteus*. Moreover, we identified karyotype variation that helps illustrate the relationships between the lupins and the extensive cytological diversity within this group. In this study we premise that lupin genomes underwent at least two rounds of fusion and fission events resulting in the reduction in chromosome number from 2*n* = 52 through 2*n* = 40 to 2*n* = 32, followed by chromosome number increment to 2*n* = 42.

## Introduction

Molecular cytogenetics offers a toolbox for integrating chromosomal data with other genetic and genomic information in order to obtain a more holistic view of genome structure and evolution. Fluorescence *in situ* hybridization (FISH) enables to study plant karyotype variation, including various chromosome rearrangements in model plants (Wolny et al., 2011; Rosato et al., 2012) and crop species, such as *Arachis* (Robledo and Seijo, 2010), *Phaseolus* (Fonseca and Pedrosa-Harand, 2013), and *Vigna* (She et al., 2015). FISH mapping is also effective approach for analyzing chromosomal remnants of polyploidization, for example detecting homeologous regions of *Glycine max* chromosomes (Lin et al., 2010) or determining the phylogenetic relationships among Brassicaceae species (Mandakova et al., 2010).

The genus *Lupinus* (lupins) belongs to the legume family (Fabaceae) that diverged from other legumes around 17–22.5 million year ago (mya) (Drummond et al., 2012). This is a monophyletic genus in the Genistoid clade, comprising ~275 species that are geographically grouped as Old World lupins (OWL) and New World lupins (NWL). The OWL, which have 12–15 crop and wild species, are centered around the Mediterranean basin and in North and East Africa (Gladstones, 1998), whereas NWL are distributed mainly in North and South America. All OWL are annual herbaceous species, with digitated leaves, in contrast to NWL that encompass both annual and perennial species with unifoliate and digitated leaves. Based on nuclear (ITS) and chloroplast (*rbc*L) DNA analyses and the genome size estimates, the OWL were divided into clades comprising two groups: smooth-seeded with four sections: Angustifolius, Albus, Luteus and Micranthus and rough-seeded with two sections: Atlanticus and Pilosus (Naganowska et al., 2003; Aïnouche et al., 2004). There are a few hypotheses regarding Old-New World group disjunction (Käss and Wink, 1997). One considering the origin of lupin diversification in Old World can be supported by the fact that unifoliolate species derived from digitated species and perennials could evolve from the annual ones (Aïnouche et al., 2004). Recently, Drummond et al. (2012) endorsed that the genus *Lupinus* derived from Old World and distinguished three evolutionary distinct lineages: (i) OWL (4.6–12.5 mya) close to the unifoliolate species from eastern North America (0.1–2.4 mya), (ii) the eastern South American species (2.3–7.1 mya) and (iii) the western NWL (5.0–13.2 mya) including the Andean and Mexican species (1.2–3.5 mya) derived from a paraphyletic group from western North America (2.1–5.5 mya).

Despite the phylogenetic trees with the support of recent genomic and transcriptomic data (Drummond et al., 2012; Cannon et al., 2015) have been created, no comparative chromosomal studies have been complemented so far. Initial cytogenetic analyses have been pertained exclusively to the estimation of genome size (0.97–2.44 pg/2C DNA), and analyses of chromosome numbers (from 2*n* = 32 to 2*n* = 52) and basic chromosome numbers (*x* = 5–7, 9, and 13) which reflect the great complexity of the organization and variation of these genomes (Pazy et al., 1977; Naganowska et al., 2003, 2006). However, in NWL the basic chromosome number (*x* = 6) is constant and all species of this group possess either 2*n* = 36 or 2*n* = 48 chromosomes, with the exception of a few representatives. Noteworthy, OWL are more heterogeneous than NWL. They include species with a similar genome size but different chromosome numbers (e.g., *L. princei* and *L. micranthus*) or, conversely, with a different DNA content but the same chromosome number (e.g., *L. princei* and *L. atlanticus*) as well as species with both the same chromosome number and genome size (*L*. *pilosus, L*. *palaestinus*).

Insight into the cytomolecular organization of their karyotypes would be helpful to identify the “footprints” of lupin chromosome evolution. *L. cosentinii*, characterized by the lowest chromosome number (2*n* = 32) and the highest number of 45S rDNA loci could be a reliable species for comparison between lupins, particularly to *L. angustifolius*, as previously studied by Hajdera et al. (2003). Moreover, *L. cosentinii* and *L. pilosus* (2*n* = 52), both belonging to rough-seeded group, could share the common ancestor with the eastern South American lupins (Käss and Wink, 1997). On the other hand, the smooth-seeded *L. micranthus* (2*n* = 52), with the highest chromosome number but the smallest genome size, known as the most widespread Mediterranean lupin, seems to be much different from all other Old World species. Due to its similarities to the rough-seeded lupins, it could represent an evolutionary line connecting both groups of OWL. *L. multiflorus* (2*n* = 36) with chromosome number comparable to *L. cosentinii* and similarity to rough-seeded species can be studied as a potential tie between NWL and OWL (Maciel and Schifino-Wittmann, 2002).

*L. angustifolius* has a relatively well-developed research infrastructure, such as genetic maps (Boersma et al., 2008; Nelson et al., 2010; Kroc et al., 2014), cytogenetic resources (Naganowska et al., 2003; Conterato and Schifino-Wittmann, 2006; Lesniewska et al., 2011) and two genomic BAC libraries (Kasprzak et al., 2006; Gao et al., 2011). Based on available (cyto) genomic data, the *L. angustifolius*-derived sets of BACs have been comparatively mapped by FISH on the chromosomes of six lupins, including a crop *L. angustifolius* and its wild botanical form *L. cryptanthus*, wild species (*L. micranthus, L. pilosus, L. cosentinii*) as well as *L. multiflorus*, as the outgroup. Here we compared the karyotypes of these species and inferred on their variation, opening the question about the relationships between the lupins at the chromosomal level.

## Materials and methods

### Plant material

We have chosen the species that belong to different sections and are quite distinct in terms of their chromosome numbers, genome size and number of rDNA loci. Some details about their taxonomy and basic cytogenetic characteristics are provided in Table 1. They originate from diverse environmental habitats: *L. cryptanthus* is considered as a subspecies of *L. angustifolius* with a similar ecology and geographical distribution; *L. micranthus* (lesser hairy blue lupin) is distributed evenly across the entire Mediterranean; *L. pilosus* (greater hairy blue lupin) occurs natively in the north-eastern Mediterranean; *L. cosentinii*, which is known as sandplain lupin, is found in the coastal region of the western Mediterranean and Morocco; and *L. multiflorus* is a species from eastern South America (Gladstones, 1998).

**Table 1:** **Key taxonomical and cytogenetic characteristics of studied *Lupinus* species**.

**Group**	**Section**	**Species**	**Accession**	**Chromosome number (2*n*)**	**Genome size (pg/2C DNA)**	**rDNA loci/haploid genome**	
						**5S**	**45S**
Smooth-seeded	Angustifolius	*L. angustifolius*	cv. “Sonet”^*^	40	1.89	1	1
		*L. cryptanthus*	96361^*^	40	1.86	2^^^	2^^^
	Micranthus	*L. micranthus*	98552^*^	52	0.98	1	1
Rough -seeded	Pilosus	*L. pilosus*	98653^*^	42	1.36	1^^^	2^^^
	Atlanticus	*L. cosentinii*	98452^*^	32	1.42	1	3
	NWL	*L. multiflorus*	508613^**^	36	1.86	1^^^	1^^^

* *Polish Lupinus Gene Bank, Breeding Station Wiatrowo, Poznan Plant Breeders Ltd., Poland*.

** *U.S. Department of Agriculture, USA*.

^ *Data obtained by Susek K. and Bielski W., others by Naganowska and Zielińska (2002)*.

### Somatic chromosome preparation

Mitotic metaphase chromosomes were obtained from the root-tip meristems following the procedure described by Lesniewska et al. (2011) with the minor modifications that were imposed by interspecific differences. Germination was accelerated by placing seeds in tap water at 25°C for 4 h. After careful scarification of the coat of the rough-seeded species, seeds were transferred onto moistened filter paper in petri dishes at 25°C. In order to accumulate cells at metaphase and ensure optimal chromosome condensation, both the primary and lateral roots, 1.5–2.0 cm in length, were excised and treated with ice-cold (2–3°C) water for 20–24 h, according to the species-specific requirements. Then, the entire roots were fixed in a freshly prepared 3:1 ethanol:glacial acetic acid mixture and stored at −20°C until used. The roots were digested in an enzyme solution comprising 40% (v/v) pectinase (Sigma-Aldrich), 3% (w/v) cellulase (Sigma-Aldrich) and 1.5% (w/v) cellulase “Onozuka R-10” (Serva) for 1.5 h for the primary roots and 1 h for the lateral roots, both at 37°C. Chromosome preparations were made from dissected meristematic tissue in a drop of 50% acetic acid on slides and frozen at −80°C. Finally, the preparations were postfixed in ice-cold 3:1 ethanol:glacial acetic acid, dehydrated in absolute ethanol for 30 min and air-dried. To localize tested BACs, at least fifty plants of each species were used for FISH experiments and about 500 full chromosome complements (cells) of each species for all used BACs, which gives about 30 cell per BAC were analyzed.

### Probe origin and labeling

The set of 16 BAC clones was comparatively mapped in cross-species FISH experiments. Twelve “single-locus”/“unique” (i.e., hybridizing to one locus only in the *L. angustifolius* genome; indicated as “U”) BAC clones (Lesniewska et al., 2011), were adopted for the present research and referred to as “S” BACs. These clones originated from the genomic BAC library created by Kasprzak et al. (2006) and were linked to the following linkage groups (NLL) of *L. angustifolius*: S44J16—NLL 06; S111G03, S136C16 and S3B18—NLL08; S84D22, S111B08, S142C04 and S142D13—NLL17; the four remaining clones were not associated to any linkage group. Additionally, four “single-locus” BAC clones (referred to as “T” BACs) from the nuclear genome BAC library of *L. angustifolius* cv. “Tanjil” (Gao et al., 2011), were also used.

BAC DNA was isolated from single *Escherichia coli* colonies using a QIAprep Spin Miniprep Kit (Qiagen) and subsequently used for labeling or sequencing. The quality and size of TBACs were estimated by pulsed field gel electrophoresis (PFGE) according to Lesniewska et al. (2011). BAC DNA was labeled by nick-translation mix (Roche Diagnostics) either with digoxygenin-11-dUTP (Roche Diagnostics), tetramethylrhodamine-5-dUTP (Roche Diagnostics) or biotin-16-dUTP (Roche Diagnostics). After precipitation in ethanol, the probes were reconstituted in 12 μl of an EB buffer.

### Comparative fluorescence *in situ* hybridization

The FISH procedure followed Jenkins and Hasterok (2007) with modifications as described by Lesniewska et al. (2011) and kinetic adjustments to complement the specific and reliable cross-species FISH reaction. In general, the slides were pre-treated with RNase (100 μg/ml) in a 2 × SSC buffer at 37°C for 1 h and washed 3 times in 2 × SSC at room temperature (RT). For slides that contained large amounts of cytoplasm, a wash in 0.01 M HCl for 2 min followed by pepsin in an HCl solution (5 μg/ml) treatment for 5–8 min was carried out. Next, the slides were washed in H_2_O for 2 min and 3 × 5 min in 2 × SSC at RT. After postfixing in 10% formalin followed by several washes in 2 × SSC, the slides were dehydrated in an ethanol series and air dried. The hybridization mixture consisted of 50% deionized formamide, 10% dextran sulfate, 2 × SSC, 0.5% SDS, and 75–200 ng probe per slide. Probes were predenatured at 90°C for 9 min and then, after applying the mixture to the slides, denatured together with the chromosome material at 73–75°C depending on individual, species-specific adjustments for 5 min and allowed to hybridize in a humid chamber at 37°C for at least 20 h. After hybridization the slides were washed in 10% deionized formamide in 2 × SSC at 37°C, which is the equivalent of 55% stringency (Schwarzacher and Heslop-Harrison, 2000). Immunodetection of the digoxigenated probes was performed with fluorescein isothiocyanate (FITC)-conjugated antidigoxigenin primary antibodies (Roche Diagnostics) for 2 h followed by the secondary immunodetection using FITC-conjugated rabbit anti-sheep antibody (Life Technologies) for an additional 1.5 h. Immunodetection of the biotinylated probes was carried out with a streptavidin-tetramethylrhodamine conjugate (Life Technologies). After final washes in Tween20/4 × SSC and ethanol dehydration, air dried slides were mounted in Vectashield (Vector Laboratories) that contained 2.5 μg/ml DAPI. All images were acquired using an F-View monochromatic camera attached to an Olympus BX-60 epifluorescence microscope, tinted in Wasabi (Hamamatsu Photonics) and superimposed using Micrografx (Corel) Picture Publisher 10 software.

### BAC sequencing and sequence analysis

The whole BAC DNA was subjected for sequencing by PacBio. Two libraries, each comprising 6 BACs, were prepared. Genomic fragments of BACs with similar chromosomal localization, were barcoded. Each sample was purified using AMPure® PB magnetic beads and quantified on Life Technologies Qubit® 2.0. Then, samples were pooled with equimolar quantities. BACs were sheared to target insert size with g-Tube from the Covaris®, purified again using AMPure® PB magnetic beads and verified on an Agilent Bioanalyzer DNA Chip. Subsequently, the library was prepared following PacBio guidelines. Then each end-repair for each sample was performed, followed by ligation of the barcoded hairpin adapters, created accordingly to the PacBio guidelines. Libraries were loaded onto the SMRT cells with the assistance of the MagBead stations and sequenced on the PacBio RS II system. Both libraries were sequenced using P6/C4 chemistry. The HGAP3 protocol, implemented in the SMRT Analysis, was employed for the *de novo* assembly of the BAC sequences. The procedure consisted of generation of long preassembled reads with improved consensus accuracy, assembly of the genome through overlap consensus accuracy using Celera Assembler, and genome polishing with Quiver algorithm (Chin et al., 2013).

The repetitive DNA present in BACs was identified by searches for similarity to sequences in RepBase repeat database, using the RepeatMasker ver 4.0.5 and Censor (Kohany et al., 2006). Whole BAC inserts were queried to *L. angustifolius* whole genome shotgun (wgs) using nucleotide BLAST algorithm (http://www.ncbi.nlm.nih.gov/). BAC sequences were also imported into Geneious 8.1.6. and mapped to reference *L. angustifolius* sequence obtained by Yang et al. (2013).

## Results

### Chromosome markers for lupins

The exploitation of *L. angustifolius* BAC-based chromosome markers as probes for FISH led to the establishment of the first set of markers (clones mapped to one locus only; U) for the following species: *L. cryptanthus* (eleven BACs, 69% of all of the clones), *L. micranthus* (five BACs, 31%), *L. pilosus* (four BACs, 25%), and *L. cosentinii* (three BACs, 19%). In order to obtain the chromosome markers for *L. multiflorus*, we used all these clones, of which, only two were “unique” (Table 2). Interestingly, despite the distant relationships, the *L. angustifolius*-derived BACs effectively hybridized to *L. multiflorus* chromosomes.

**Table 2:** **Summary of *L. angustifolius* BAC clones used for the cross-species BAC-FISH**.

**Lang**	**BAC ID**	***L. cryptanthus* (2*n* = 40)**	***L. micranthus* (2*n* = 52)**	***L. pilosus* (2*n* = 42)**	***L. cosentinii* (2*n* = 32)**	***L. multiflorus* (2*n* = 36)**
		**BAC-FISH signal**				
06	S44J16	U	R	N/D	N/D	R
08	S84D22	U	U+	U	U+	U
	S111B08	U	U	U+	U+	R
	S142C04	U	U	R	R	R
	S142D13	U	R	R	R	R
17	S3B18	U	U	U	U	U+
	S111G03	R	U	U	U	R
	S136C16	U	U	U	U	U
Unlinked	S1M23	U	U+	U+	R	R
	S2B03	U	R	U+	U+	U+
	S6E05	U	R	R	R	R
	S8C03	U	U+	R	R	R
	T108A01	R	R	R	R	R
	T109I09	R	R	R	R	R
	T120C20	R	R	R	R	R
	T124G17	R	R	R	R	R

*Lang, chromosome of L. angustifolius corresponding to linkage group, U, “unique” signal; U+, “unique” with minor signals; R, “repetitive” signals on many chromosomes; N/D, not detected*.

**Figure 1:**
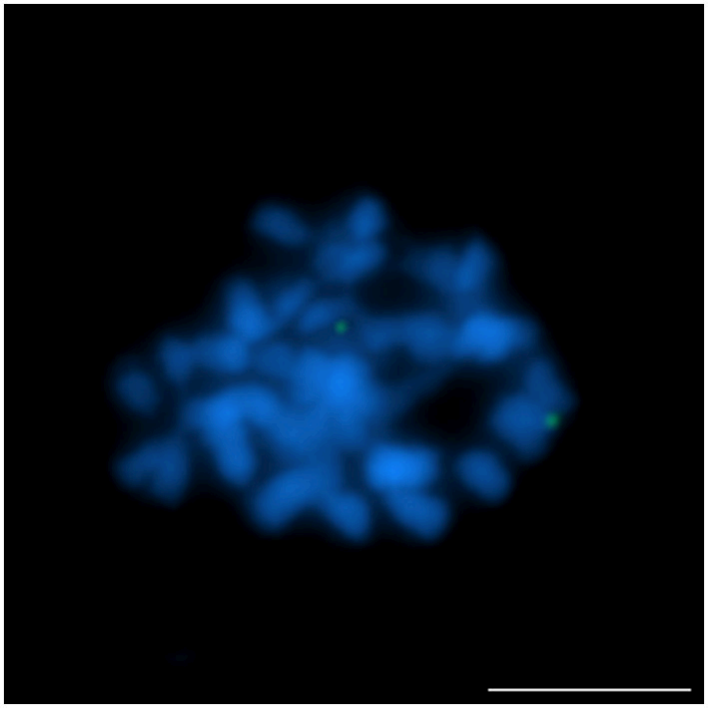
**Comparative FISH using BAC clone S84D22 (green) from the *L. angustifolius* chromosome Lang08 hybridizing to *L. multiflorus* chromosomes**. Chromosomes visualized in blue, scale bar: 5 μm.

**Figure 2:**
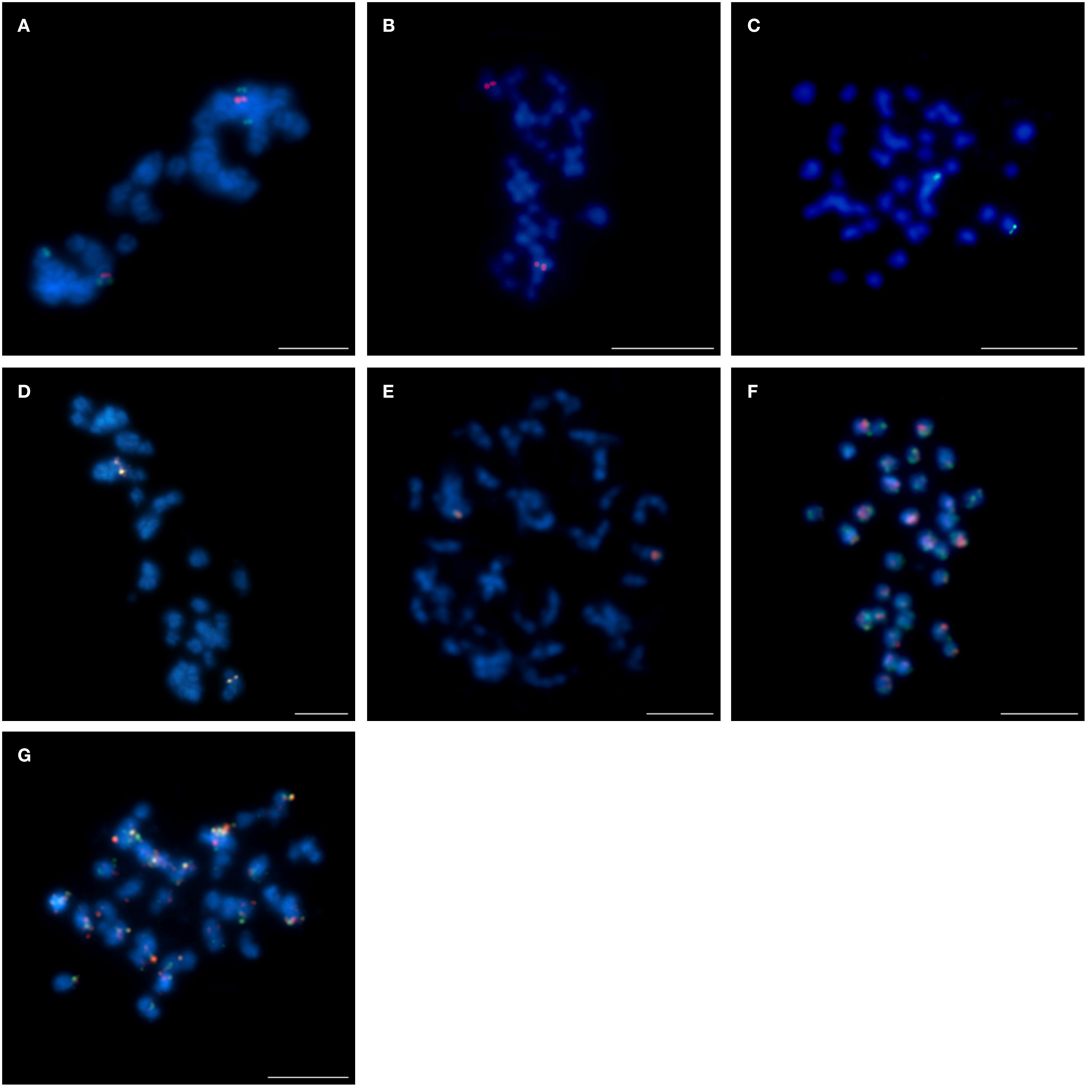
**Comparative FISH using BAC clones from the *L. angustifolius* cv. “Tanjil” genomic library hybridizing to the chromosomes of: (A) *L. angustifolius* cv. “Tanjil,” (B,C) *L. angustifolius* cv. “Sonet,” T108A01 (red) and T109I09 (green); (D) *L. angustifolius* cv. “Tanjil,” (E) *L. angustifolius* cv. “Sonet,” T124G17 (red) and T120C20 (green); (F) *L. cosentinii*, T108A01 (red) and T109I09 (green); (G) *L. cosentinii*, T124G17 (red) and T120C20 (green)**. Chromosomes visualized in blue, scale bar: 5 μm.

Four clones (S84D22, S111B08, S3B18, and S136C16) were “unique” or “unique” with minor/ additional signals (indicated as U+; Table 2) in *L. cryptanthus, L. micranthus, L. pilosus*, and *L. cosentinii*. Two BACs, S84D22 and S111B08, gave additional signals in *L. cosentinii* but S84D22 only in *L. micranthus* and S111B08 only in *L. pilosus*. In *L. multiflorus* all but S111B08 were “unique,” and therefore provided chromosome markers for this species (as an example S84D22, Figure 1). Other clones were either characterized as “repetitive” (with a dispersed signal over chromosomes; R) or U/U+ in particular species. In *L. micranthus, L. pilosus* and *L. cosentinii*, we detected three BACs that hybridized as U+. We identified five BACs that behaved like “repetitive” for *L. cryptanthus*, eight for both *L. micranthus*, and *L. pilosus* and nine in the case of *L. cosentinii*. Twelve clones gave “repetitive” signals on chromosomes in *L. multiflorus*. Interestingly, all four TBACs that mapped as U in both cultivar varieties of *L. angustifolius* (Figures 2A–E) were “repetitive” in all of the analyzed species (as an example, *L. cosentinii* Figures 2F,G). Therefore, the TBACs could not be utilized as reliable markers for tracking chromosome rearrangements. Among all 16 clones the BAC S44J16 was not detected at all in *L. pilosus* and *L. cosentinii* (indicated as N/D in Table 2).

### Chromosomal variation detected by BAC-FISH

**Figure 3:**
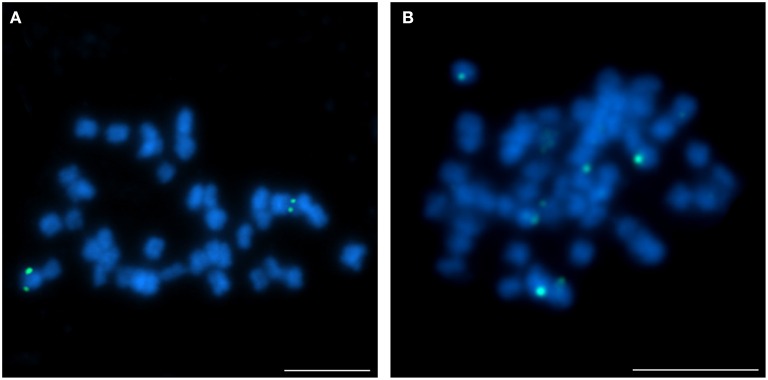
**Comparative FISH using BAC clone S44J16 (green) from the *L. angustifolius* chromosome Lang06 hybridizing to (A) *L. cryptanthus* and (B) *L. micranthus* chromosomes**. Chromosomes visualized in blue, scale bar: 5 μm.

The hybridization of 16 clones to chromosomes of studied species revealed various patterns. Thus BAC-FISH approach indicated that some structural changes have occurred during the evolution of *Lupinus* karyotypes. The *L. angustifolius* chromosome Lang06 was represented by one marker, BAC S44J16, which was also “unique” for *L. cryptanthus* (Figure 3A), the species considered to be more closely related to *L. angustifolius* than the others. However, the same clone was “repetitive” in *L. micranthus* (Figure 3B) and was not detected at all in *L. pilosus* and *L. cosentinii* (data not shown).

**Figure 4:**
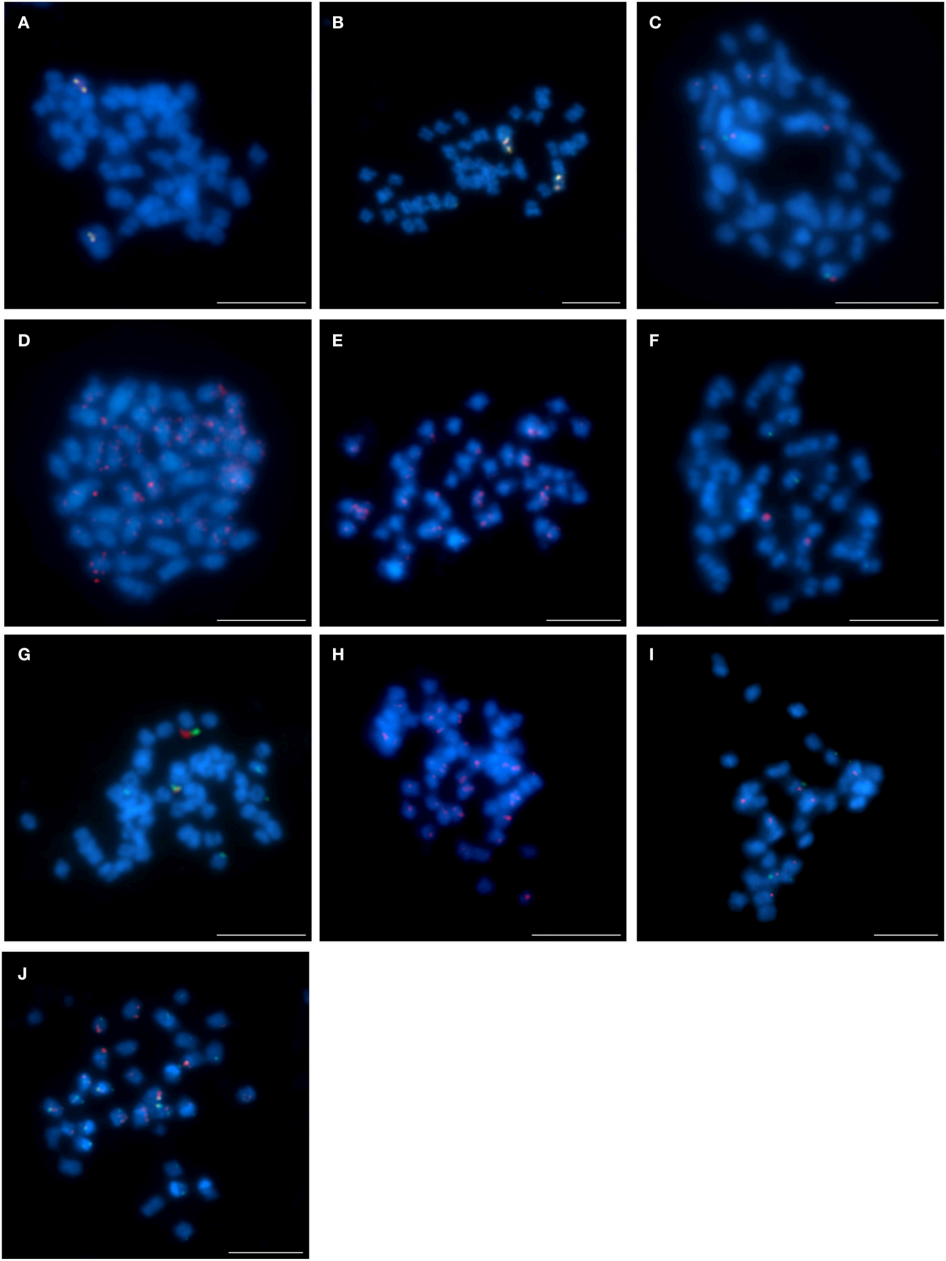
**Comparative FISH using BAC clones from the *L. angustifolius* chromosome Lang08 hybridizing to the chromosomes of: (A,B) *L. cryptanthus*, S84D22 (green) with S111B08 (red) and S142C04 (green) with S142D13 (red), respectively; (C) *L. pilosus*, S84D22 (green) with S111B08 (red), (D,E) *L. pilosus*, S142C04 (red) and S142D13 (red), respectively; (F,G) *L. micranthus*, S84D22 (green) with S111B08 (red) and S84D22 (green) with S142C04 (red), respectively; (H) *L. micranthus*, S142D13 (red); (I,J) *L. cosentinii*, S84D22 (green) with S111B08 (red) and S142C04 (red) with S142D13 (green), respectively**. Chromosomes visualized in blue, scale bar: 5 μm.

The pattern of the localization of the four clones (S84D22, S111B08, S142C04, and S142D13) from the chromosome Lang08 illustrated an interspecific structural variation among the lupins. Comparative BAC-FISH mapping revealed that all BACs in *L. cryptanthus* (Figures 4A,B), when mapped pairwise, localized similarly to what was observed in *L. angustifolius* (Lesniewska et al., 2011). This indicates that this genomic region has not undergone any structural rearrangements, at least at the resolution afforded by this approach. The pattern of the localization of clones S84D22 and S111B08 in the same pair of chromosomes was also found in *L. pilosus* (Figure 4C), whereas clones S142C04 and S142D13 (Figures 4D,E, respectively) hybridized to multiple sites along the entire chromosomes, which may suggest that various changes may have occurred within this chromosome region. In *L. micranthus* (Figure 4F), S84D22 and S111B08 were mapped on different pairs of chromosomes, whereas clones S84D22 and S142C04 localized on the same chromosome pair, excluding few minor signals that were observed for S84D22 only (Figure 4G), and S142D13 hybridized to multiple chromosomal sites (Figure 4H). The set of all Lang08-derived BACs hybridized only to a few (Figure 4I; S84D22 and S111B08) or many (Figure 4J; S142C04 and S142D13) chromosomes in *L. cosentinii*.

**Figure 5:**
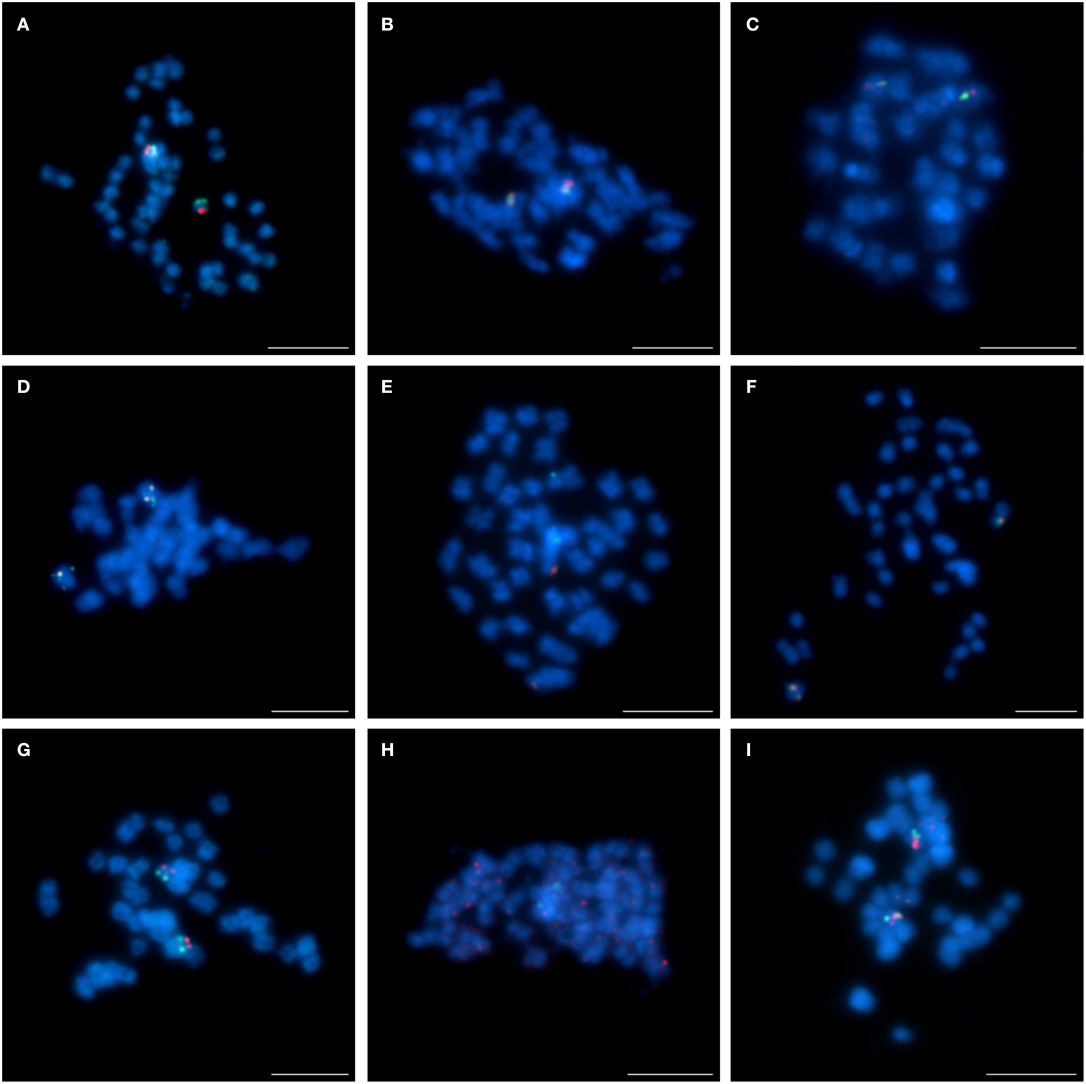
**Comparative FISH using BAC clones from the *L. angustifolius* chromosome Lang 17 hybridizing to the chromosomes of: (A,B) *L. micranthus*; (C,D) *L. cosentinii*; (E,F) *L. pilosus*; (G,H) *L. cryptanthus*; (I) *L. multiflorus*; (A,C,E,G,I) S3B18 (red) with S136C16 (green) and (B,D,F,H), S111G03 (red) with S136C16 (green), respectively**. Chromosomes visualized in blue, scale bar: 5 μm.

The application of the clones from chromosome Lang17 demonstrated other chromosome variation. Interestingly, all three BACs (S3B18, S111G03, and S136C16) mapped on one pair of chromosomes in *L. micranthus* (Figures 5A,B) and *L. cosentinii* (Figures 5C,D), exactly as it was shown by Lesniewska et al. (2011) for *L. angustifolius*, in which the BAC S3B18 was located in one arm and S136C16 and S111G03 occupied the opposite arm. In *L. pilosus* (Figure 5E), the clones S3B18 and S136C16 hybridized with two different chromosome pairs, thus illustrating rearrangements in comparison to *L. angustifolius*, as well as *L. micranthus* and *L. cosentinii*. However, clones S111G03 and S136C16 hybridized to only one chromosome pair in *L. pilosus* (Figure 5F). In *L. cryptanthus*, the clones S3B18 and S136C16 were identified in the opposite arms of one chromosome pair (Figure 5G) but S111G03 hybridized to many sites along the chromosomes (Figure 5H), as was also observed in *L. multiflorus* (Figure 5I; S3B18 and S136C16).

**Figure 6:**
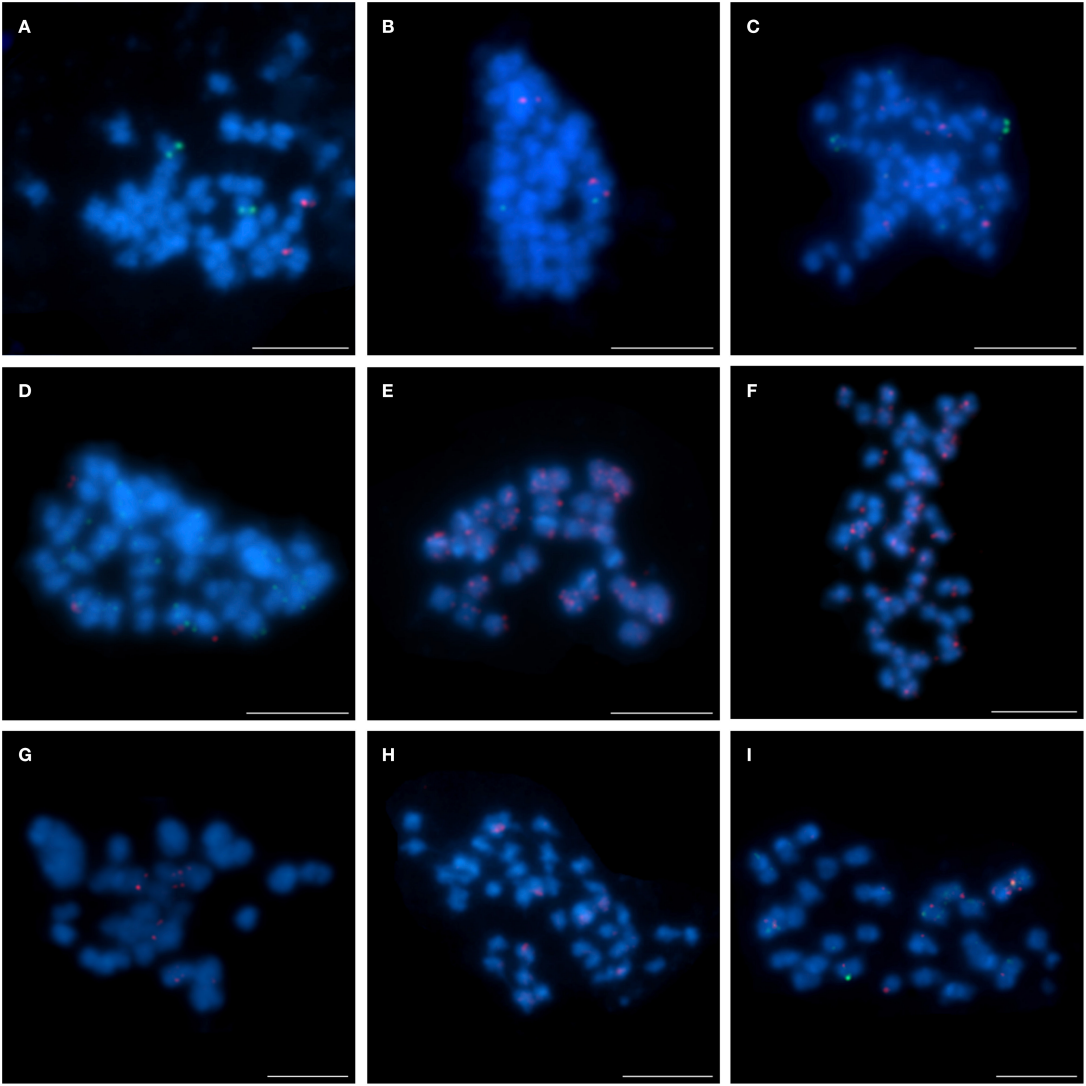
**Comparative FISH using BAC clones from unlinked chromosome of *L. angustifolius* hybridizing to the chromosomes of: (A,B) *L. cryptanthus* S1M23 (red) with S8C03 (green) and S2B03 (red) with S6E05 (green), respectively; (C) *L. micranthus* and (D) *L. pilosus*, S1M23 (red) with S8C03 (green); (E) *L. cosentinii*, S1M23 (red); (F) *L. micranthus*, (G) *L. multiflorus* and (H) *L. pilosus*, S2B03 (red); (I) *L. cosentinii*, S2B03 (red) with S6E05 (green)**. Chromosomes visualized in blue, scale bar: 5 μm.

The four remaining chromosome-specific BACs (S1M23, S2B03, S6E05, and S8C03), as yet not assigned to any specific *L. angustifolius* linkage group, were also subjected to comparative BAC-FISH. We found that all these clones displayed “unique” signals in *L. cryptanthus* (as an example of S1M23 and S8C03, Figure 6A and S2B03 and S6E05, Figure 6B). Nonetheless, BAC S1M23 was found to be U+ in *L. micranthus* (Figure 6C, red) and *L. pilosus* (as an example, Figure 6D, red) and R in *L. cosentinii* (Figure 6E). The clone S2B03 was identified as R in *L. micranthus* (as an example, Figure 6F) but as U in *L. multiflorus* (Figure 6G), U+ in *L. pilosus* (Figure 6H) and *L. cosentinii* (as an example, Figure 6I, red). Two other clones (S6E05 and S8C03), which were “unique” for *L. cryptanthus*, hybridized to multiple sites along the chromosomes in *L. pilosus* (as an example for S8C03 Figure 6D, green) and *L. cosentinii* (an example for S6E05 Figure 6I, green) but they gave R and U+ signals in *L. micranthus*, respectively (as an example for S8C03 Figure 6C, green).

### BAC sequence analysis

The set of 12 SBACs, examined as markers to identify the variation in lupin karyotypes was subjected for sequencing. All sequences were deposited in GenBank (KX298063-KX298074). The sizes of all TBACs were estimated as about 100 kbp and along with the sizes of SBACs were listed in Supplementary Table 1.

**Figure 7:**
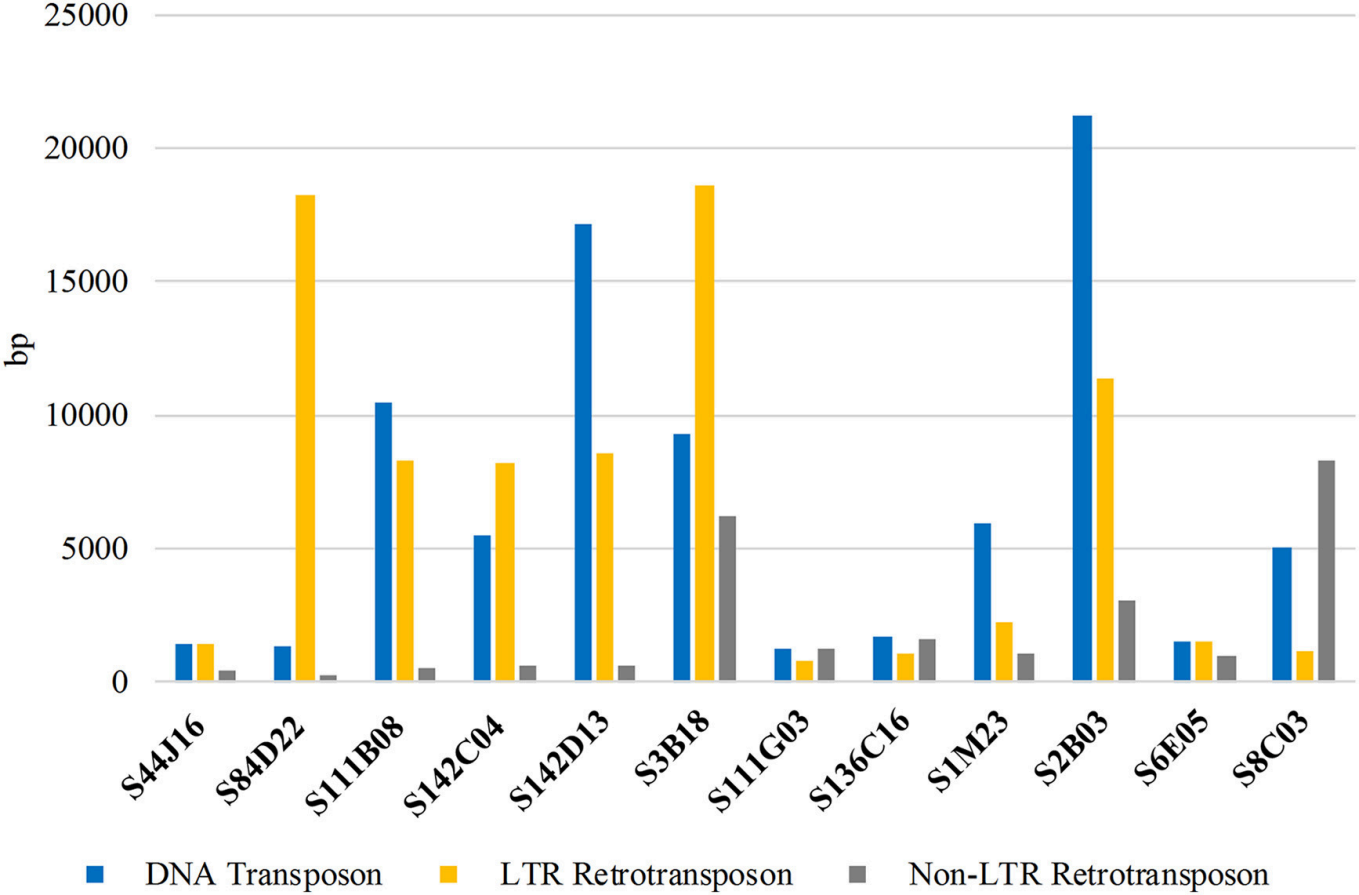
**The sum of the length (bp) of different types of repetitive DNA sequences in BACs used for comparative studies among lupins**.

To provide the insight into the repetitive sequence content of BACs used for comparative karyotyping we identified the sum of the length of the sequences with homology to certain types of transposable elements (TEs) in the databases (NCBI, GIRI). The LTR as well as non-LTR transposons (Figure 7; Supplementary Table 1) were abundantly represented. The total length of DNA transposon and LTR retrotransposons in all sequenced BACs was similar, 81,833 bp and 81,486 bp, respectively. The sum of non-LTR retrotransposons was estimated as 24,831 bp. The clones S2B03 and S142D13 had the highest (21,232 bp and 17,121 bp) sum of the length of DNA transposons, representing 28.3% and 14.2% of the total BAC, respectively. The DNA transposons such as *EnSpm/CACTA, Helitron*, and *hAT* were also identified in all sequenced clones. We observed that the elements like *Mariner/Tc1, MuDR*, and *Politon* were ampler than *Harbinger, Kolobok* and *Transib*. On the other hand, we found that some TEs (*Crypton* and *Merlin*) were not ubiquitous but were present only in some clones (S3B18, S111G03, S136C16, S1M23, and 2B03). The smallest amount of DNA transposons was about 1200–1300 bp and was found in S111G03 and S84D22. The LTR retrotransposons, mainly *Gypsy* and *Copia* elements were identified in all clones but at different lengths, ranging from 18,617 bp (S3B18) to 801 bp (S111G03). Interestingly, the clone S84D22 displayed the highest disproportion between DNA transposons and LTR retroelements. We also revealed that non-LTR retroelements were the least represented ranging from 8285 bp (S8C03) to 240 bp (S84D22).

Although the draft of the *L. angustifolius* cv. “Tanjil” genome is still fragmented into numerous short scaffolds (with the average scaffold length 2511 bp), we made the effort to succeed in aligning the clones mapped in similar position on chromosomes. Most of the clones provided alignments of 100% sequence identity value and were considered as proper assignment to scaffolds, with the exception of clone S111B08, where the identity value has been assigned below 92%. The contig (~28 kbp) consisting of S136C16, S111G03 and the scaffold AOCW01144278 illustrated that these clones corresponded to the same region of ~60 kbp in *L. angustifolius* (Lang17). The clones representing Lang08: S84D22, S111B08, 142C04, and 142D13 were established in the contig of 160 kbp and have been mapped to the scaffold AOCW01048841.

## Discussion

### Chromosome markers for lupin karyotype studies

FISH is an arduous and time-consuming but useful method that enables robust chromosome markers for many plant species to be obtained and genome maps to be effectively integrated as shown, e.g., in *Phaseolus* (Pedrosa-Harand et al., 2009; Bonifácio et al., 2012). Similar analyzes to be done for the lupins represent a greater challenge, also due to considerably less favorable organization of their genomes, with high and diverse chromosome numbers that vary from 2*n* = 52 through 2*n* = 40 (in *L. angustifolius*) to 2*n* = 32.

In this study, the highest number of seven successfully tagged chromosomes was achieved for *L. cryptanthus* (2*n* = 40). In contrast, in *L. cosentinii* (2*n* = 32) only three chromosomes were identified. The patterns of BAC localization in lupin chromosomes are differentiated by various chromosomal changes. Some BACs allowed to identify genomic regions that have underwent multiple changes. The good example is chromosome Lang08 that can be linked to two chromosomes of *L. micranthus* and one chromosome of *L. pilosus* and *L. cosentinii*. The karyotype similarity between *L. pilosus* and *L. cosentinii* can also be observed in case of other BACs, with the exception of S3B18 (Lang17) and S1M23. The *L. micranthus* chromosomes seemed to much differ from chromosomes of other analyzed species, which can indicate that the karyotype of this species underwent some specific changes during the evolution.

The development of BAC libraries for *L. angustfolius* opened new opportunities for comparative cytomolecular studies of lupin genomes. We demonstrated that *L. angustifolius*-derived BAC markers can be a valuable and effective tool for the identification of lupin chromosomes. To our knowledge, these are the first chromosome-specific markers for both crop and wild lupins. It is successful outcome, especially considering that rDNA markers, though very effective in many other plants, e.g., Brassicaceae (Hasterok et al., 2006), Apiaceae (Iovene et al., 2008) and *Prospero* (Jang et al., 2013), have proven to be of very little use in the *Lupinus* genus (Naganowska and Zielińska, 2002; Hajdera et al., 2003). Almost all lupins possess only one 5S rDNA locus per haploid genome, with the exception of *L. angustifolius* cv. “Emir,” which has four loci per haploid genome (Hajdera et al., 2003) and *L. cryptanthus* possessing two loci per haploid genome (Table 1). 45S rDNA is carried by only one pair of chromosomes in *L. angustifolius, L. micranthus* and *L. multiflorus*, two and three pairs in *L. pilosus* and *L. cosentinii*, respectively (Naganowska and Zielińska, 2002; Naganowska et al., personal communication). Moreover, our BACs, which have been tested for other taxa, are promising in constituting the basis for the assignment of the chromosomal and genetic maps in other important lupins, such as the crops *L. albus* (Phan et al., 2007) and *L. luteus* (Parra-González et al., 2012). Although it could be expected that high numbers of identified chromosomes are obtained for the lupins that are more closely related with *L. angustifolius* (Jiang and Gill, 2006), in this paper we present the feasibility and utility of BAC-FISH approach also to the New World lupin representatives, i.e., *L. multiflorus* that evolved 2.5 mya after *L. angustifolius* (Drummond et al., 2012). The presented BAC-FISH system could pave the straight way for further comparative karyotype investigation among lupins, leading eventually to the reconstruction of their ancestral karyotype, as it was shown for *Solanum* (Szinay et al., 2012). Besides that the BAC clones have become efficient markers at cytological level, they also serve as a template for development of genetic markers, e.g., SSR (Long et al., 2013; Blair et al., 2014). BES-SSR markers could be effective for comparative mapping, especially by construction of genetic maps of lupins as it was shown for other legumes (Bohra et al., 2011; Thudi et al., 2011).

### Karyotype variation in lupins

**Figure 8:**
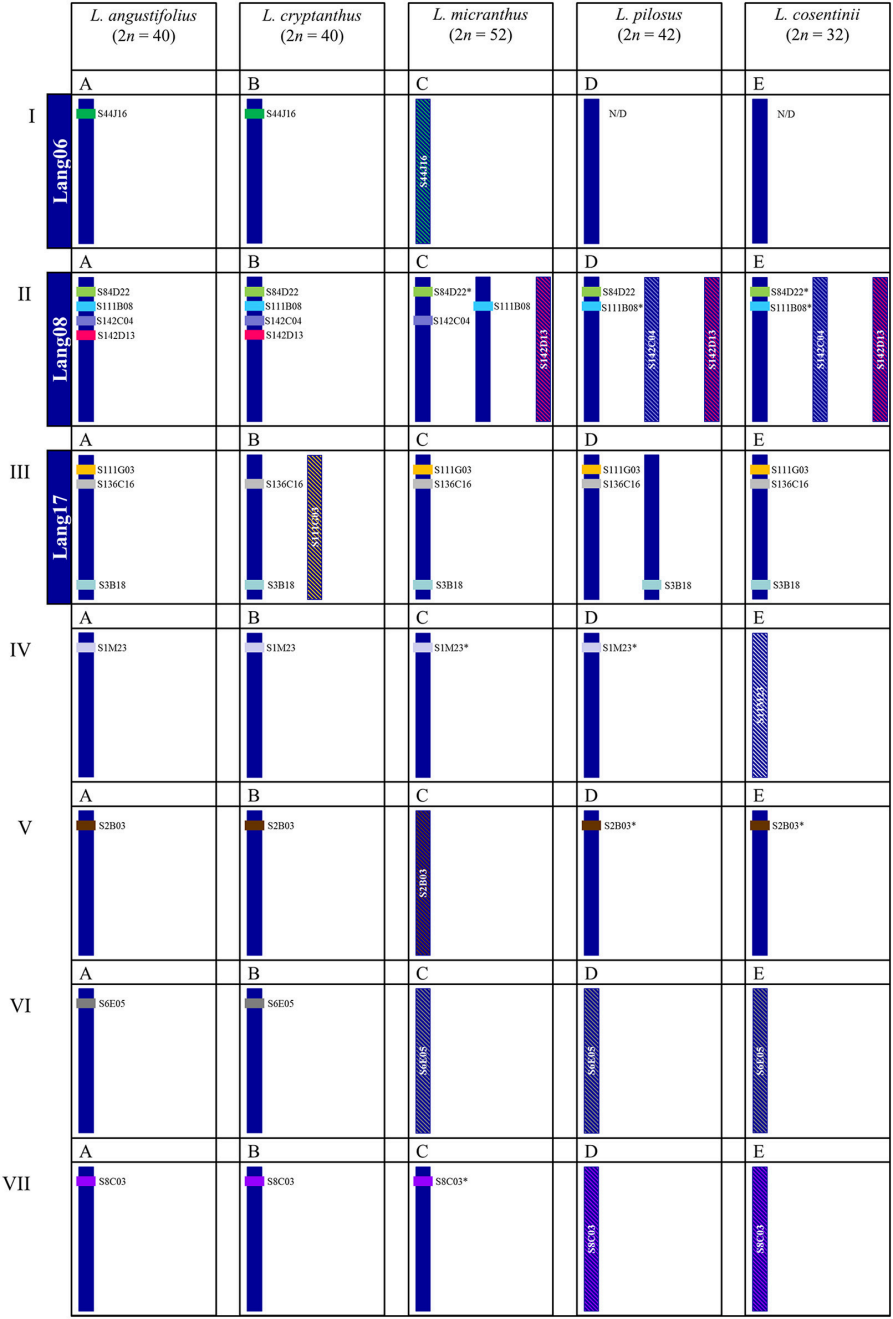
**Schematic overview of chromosomal rearrangements among the lupins identified by cross-species BAC-FISH**. Roman numerals correspond to the chromosomes of a reference species, *L. angustifolius* and Latin letters above each schematic chromosome(s) are assigned to species used in this study. **(A)** *L. angustifolius*, **(B)** *L. crypthantus*, **(C)** *L. micranthus*, **(D)** *L. pilosus*, **(E)** *L. cosentinii*. Lang06, 08 and 17 indicate linkage groups of *L. angustifolius* (Lesniewska et al., 2011). BAC clones mapped were indicated by color code, e.g., the clone S44J16 is shown in green. Various patterns of BAC-FISH signal(s) are presented as: 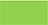 “unique” signal, 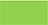 ^*^ “unique” with minor additional signals, 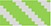 “repetitive” signals on many chromosomes.

Comparative mapping using clones derived from one species enables to identify homeologous regions in chromosomes of related species (Xiong and Pires, 2011) and shed some light on their evolution (Lin et al., 2010). Based on the different types (U, U+, R) of BACs, we observed several distinct patterns of rearrangements as compared to *L. angustifolius* (Figure 8IA vs. Figures 8ID,E). The least differences in the karyotype structure were observed between *L. angustifolius* and *L. cryptanthus*, which most likely can be explained by the fact that *L. cryptanthus* is considered to be a wild form of *L. angustifolius* (Naganowska et al., 2003). The rearrangements involving other chromosomes (*L. angustifolius*-like Lang08 and Lang17) in all studied wild lupins imply multiple and complex structural reshufflings that occurred in their karyotypes during evolution (Figure 8IIA vs. Figures 8IIC–E and Figure 8IIIA vs. Figures 8IIIC–E). The presence of chromosome fusion and fission rearrangements is postulated to be one of the important forces that shape the structure of plant karyotypes, as demonstrated by FISH in *Brachypodium* (Wolny et al., 2011) *Cardamine* (Mandáková et al., 2013) and *Phaseolus* (Fonseca et al., 2016). This phenomenon has been extensively studied in grasses, cereals in particular (Salse and Feuillet, 2011), and also in other plants, including some legumes (Murat et al., 2015). Such events cannot be ruled out also as one of the drivers of lupin karyotype evolution. Phylogenetic trees established with various nuclear (*GPAT*1, *GPAT*2, ITS1+2, *LEGCYC*1A, *LEGCYC*1B) and chloroplast (*mat*K, *rbc*L, *trn*L intron, *trn*L–*trn*F, *trn*S–*trn*G, and *trn*T–*trn*L) DNA sequences exemplified that OWL were divided about 8.0 mya into two main groups, the first one including older *L. micranthus*/*L. angustifolius* and more diverse group but with younger *L. cosentinii* and *L. pilosus*. Along with phylogenetic data (Drummond et al., 2012) it is tempting to speculate that the karyotype with 52 chromosomes corresponding to the *L. micranthus* one, could undergo multiple fusions (~7.5 mya) leading to the karyotype with 40 chromosomes, such as in *L. angustifolius* (~4.5 mya) followed by further reduction to 32 chromosomes (*L. cosentinii*, ~4.0 mya). On the other hand, fissions may have also occurred leading to the karyotypes with 32 chromosomes that led to the appearance of other karyotypes with 42 chromosomes (*L. pilosus*, ~3.0 mya), considering that one chromosome of *L. cosentinii* (Figure 8IIE) could correspond to two chromosomes of *L. pilosus* (Figure 8IID). These multiple rearrangements could be supported by Kroc et al. (2014) and Przysiecka et al. (2015) that postulated *L. angustifolius* to undergo a duplication and/or triplication. Moreover, evolutionary trees of legumes constructed with a great number of transcriptome sequences, revealed the duplications in *L. angustifolius* and *L. polyphyllus* (Cannon et al., 2015). The authors also concluded that duplication events could take place before the divergence of NWL and OWL.

In an extensive comparative study of plants, involving such legumes as *G. max* and *Lotus japonicus*, Murat et al. (2015) hypothesized an ancestral karyotype for the Fabaceae with *n* = 21 was changed by one fission and sixteen fusions thus forming a Fabaceae intermediate karyotype with *n* = 6, which underwent the first (prepapilionoid) whole genome duplication (WGD) and formed a Papilionideae intermediate karyotype with *n* = 12. The postpapilionoid WGD then established a karyotype with *n* = 24 and further rearrangements led to a karyotype with *n* = 20, which is now exemplified by *G. max* that has the same chromosome number as *L. angustifolius*. To highlight this complexity, it was suggested that the genistoid clade is derived from an ancestor with *x* = 9 (Cannon et al., 2015).

Our study gives some cytomolecular evidence on the puzzling and complicated nature of the genome evolution of lupins and shows that some duplication processes resulted in extra copies of some BACs (e.g., S84D22, Figures 8IIA,B,D vs. Figures 5IIC,E; S111B08, Figures 6IIA–C vs. Figures 8IID,E; S1M23, Figures 8IVA,B vs. Figures 8IVC,D; S2B03, Figures 8VA,B vs. Figures 8VD,E; S8C03, Figures 8VIIA,B vs. Figure 8VIIC), the amplification of some repetitive sequences (S44J16, Figures 8IA,B vs. Figure 8IC; S142D13, Figures 8IIA,B vs. Figures 8IIC–E; S142C04, Figures 8IIA–C vs. Figures 8IID,E; S111G03, Figures 8IIIA,C–E vs. Figure 8IIIB; S1M23, Figures 8IVA–D vs. Figure 8IVE; S2B03, Figures 8VA,B,D,E vs. Figure 8VC; S6E05, Figures 8VIA,B vs. Figures 8VIC–E; S8C03, Figures 8VIIA,B vs. Figures 8VIID,E) or their elimination (S44J16, Figures 8IA–C vs. Figures 8ID,E). These examples suggest that various structural changes were acting at the chromosome level in the lupin genomes.

Implementation of probes containing significant amounts of repetitive DNA can add a lot to the survey on plant karyotype rearrangements (Jiang and Gill, 2006). The BAC-FISH probes that produced “repetitive” signals depicted several patterns of repeat distribution in the lupin genomes but mostly limited to many chromosomes with enrichment of FISH signal at the distal regions. Thus, they may give some hint which *L. angustifolius*-like region has been prone to various rearrangements within wild relatives. Increase in the amount of repeats is often positively correlated with a reduction in the chromosome number. In *L. cosentinii* we found that over 50% of *L. angustifolius*-like BACs resulted in dispersed hybridization signals, concordant with our hypothesis of a reduction in chromosome number being accompanied by an accumulation of repetitive sequences that could happen during polyploidization events (Wendel, 2015). This is in agreement with the already mentioned phylogenetic analyses using combined sequence data, which showed that *L. albus, L. micranthus* or *L. angustifolius* may be the basal among the lupins with the divergence time of ~7.5 mya and that *L. cosentinii* can be regarded as one of the youngest (Drummond et al., 2012). A corollary and possibly alternative explanation is that there was a lineage specific amplification of repeats that were present in the ancestor genome as it was demonstrated for *Gossypium* (Hawkins et al., 2006).

This is of interest that the amount of representatives belonging to two classes of TEs is comparable, which contradicts some literature data which show the LTR retrotransposons to be the most abundant in plant genomes (Domingues et al., 2012). In addition, LTR retrotransposons were also the most plentiful class of TEs, representing 10.26% of the genome estimated based on BESs sequences of *L. angustifolius* cv. “Tanjl” (Gao et al., 2011). The presence of *CACTA, Gypsy, Copia*, and *hAT* elements in all sequenced BACs underline their highly conserved origin (Langdon et al., 2003; Schmutz et al., 2010). On the other hand, some non-LTR retrotransposons (SINE elements) have not been identified in four BACs mapped on chromosome Lang08. It might be assumed that this region could experience independent evolutionary event (Deragon and Zhang, 2006). However, it should be emphasized that till lupin genomic data sequence are scarce, accurate detecting and annotating of TEs is vexatious due to their diversity, even within the genus (Hoen et al., 2015).

The various patterns of TE organization can be explained by rapid karyotype remodeling and as response to diverse environmental conditions (Wicker and Keller, 2007). However, no correlation was found between the TE composition of BAC clones and their FISH pattern in analyzed lupins. Whereas all SBACs mapped to a single locus in *L. angustifolius*, some of them revealed different hybridization patterns in wild relatives. TE description in *L. angustifolius* could be recognized as an important stage to minimize the inaccuracy of gene annotation and facilitate functional gene analyses. In the future, it could also lead to the identification of genus specific families or types of repeats within *Lupinus*.

This study is a useful starting point for more extensive comparative analyses aiming better understanding of the evolutionary mechanisms that shape the structure and composition of lupin genomes. There is no doubt that the application of greater number of markers and the inclusion of more species would lead to a more broaden view on the cytomolecular organization of lupin genomes and the evolution of their karyotypes. Lupins, which comprise both crop and wild species, exhibit a high level of genomic diversification, and thus could be a valuable model for tracking polyploidization events and their subsequent effects on genome evolution and plant adaptation (Wendel et al., 2016). Along with extended in the future genetic resources it would be useful for better understanding of the evolution of plant genomes of the Genistoid clades, such as *Ulex* (2*n* = 80, 96), *Genista* (2*n* = 48) and *Crotalaria* (2*n* = 14, 16), all of which are species with high and diverse chromosome numbers.

## Author contributions

KS: conception and design of the work, analysis and interpretation of data for work, drafting and revising the manuscript. WB: acquisition and analysis of data for the work, drafting (partially) the work. RH: interpretation of data for the work, revising the manuscript. BN, BW: revising the manuscript. KS, WB, RH, BN, BW: final approval of the manuscript to be published and agreement to be accountable for all aspects of the work in ensuring that questions related to the accuracy or integrity of any part of the work are appropriately investigated and resolved.

### Conflict of interest statement

The authors declare that the research was conducted in the absence of any commercial or financial relationships that could be construed as a potential conflict of interest.
