# Supplementary material for: A First Glimpse of Wild Lupin Karyotype Variation As Revealed by Comparative Cytogenetic Mapping
Source: Front Plant Sci. 2016 Jul 28;7:1152. doi: 10.3389/fpls.2016.01152 (PMC4964750; doi:10.3389/fpls.2016.01152)
Supplement: Supplementary file 1 [file Table1.PDF]

*Supplementary Material*

# A first glimpse of wild lupin karyotype variation as revealed by comparative cytogenetic mapping

Karolina Susek[1]*^, Wojciech Bielski[1]^, Robert Hasterok[2], Barbara Naganowska[1] and Bogdan Wolko[1]

*** Correspondence:** Karolina Susek: ksus@igr.poznan.pl

## 1 Supplementary Figures and Tables

**Supplementary Table 1** The size of BACs used for comparative mapping and their transposable element (TE) characteristics

| BAC ID | Size PFGE* / sequencing (kbp) | The length of TEs /BAC (bp) | | | TEs / BAC (%) |
|---|---|---|---|---|---|
| | | DNA transposon | LTR | non-LTR | |
| S44J16* | ~30/20 | 1448 | 1448 | 454 | 17 |
| S84D22* | ~75/86 | 1343 | 18246 | 240 | 23 |
| S111B08* | ~130/130 | 10472 | 8320 | 523 | 15 |
| S142C04* | ~120/112 | 5511 | 8202 | 5589 | 13 |
| S142D13* | ~130/121 | 17129 | 8545 | 578 | 22 |
| S3B18* | ~140/156 | 9305 | 18617 | 6189 | 22 |
| S111G03* | ~35/28 | 1206 | 0801 | 1224 | 12 |
| S136C16* | ~50/40 | 1693 | 1083 | 1630 | 11 |

| | | | | | |
|---|---|---|---|---|---|
| S1M23* | ~20/14 | 5916 | 2210 | 1106 | 66 |
| S2B03* | ~75/75 | 21232 | 11352 | 3039 | 47 |
| S6E05* | ~30/20 | 1485 | 1484 | 974 | 20 |
| S8C03* | ~50/50 | 5093 | 1178 | 8285 | 29 |
| T108A01 | ~100 | N/A | N/A | N/A | N/A |
| T109I09 | ~100 | N/A | N/A | N/A | N/A |
| T120C20 | ~100 | N/A | N/A | N/A | N/A |
| T124G17 | ~100 | N/A | N/A | N/A | N/A |
| Total length of TEs (bp): | | 81,833 | 81,486 | 24,831 | not applicable |

* BACs that sizes were published by Lesniewska et al. (2011)

N/A not analyzed (SBACs were subjected to sequencing due to their ‘unique’ (U, U+) BAC-FISH patterns in compared lupins).
